# A Bayesian computational model for online character recognition and disability assessment during cursive eye writing

**DOI:** 10.3389/fpsyg.2013.00843

**Published:** 2013-11-11

**Authors:** Julien Diard, Vincent Rynik, Jean Lorenceau

**Affiliations:** ^1^Laboratoire de Psychologie et NeuroCognition, Université Grenoble Alpes–CNRSGrenoble, France; ^2^Centre de Recherche de l'Institut du Cerveau et de la Moelle Epinière, Université Pierre et Marie Curie–Inserm–CNRSParis, France

**Keywords:** Bayesian modeling, character recognition, eye writing, man-machine interaction, gaze interaction

## Abstract

This research involves a novel apparatus, in which the user is presented with an illusion inducing visual stimulus. The user perceives illusory movement that can be followed by the eye, so that smooth pursuit eye movements can be sustained in arbitrary directions. Thus, free-flow trajectories of any shape can be traced. In other words, coupled with an eye-tracking device, this apparatus enables “eye writing,” which appears to be an original object of study. We adapt a previous model of reading and writing to this context. We describe a probabilistic model called the Bayesian Action-Perception for Eye On-Line model (BAP-EOL). It encodes probabilistic knowledge about isolated letter trajectories, their size, high-frequency components of the produced trajectory, and pupil diameter. We show how Bayesian inference, in this single model, can be used to solve several tasks, like letter recognition and novelty detection (i.e., recognizing when a presented character is not part of the learned database). We are interested in the potential use of the eye writing apparatus by motor impaired patients: the final task we solve by Bayesian inference is disability assessment (i.e., measuring and tracking the evolution of motor characteristics of produced trajectories). Preliminary experimental results are presented, which illustrate the method, showing the feasibility of character recognition in the context of eye writing. We then show experimentally how a model of the unknown character can be used to detect trajectories that are likely to be new symbols, and how disability assessment can be performed by opportunistically observing characteristics of fine motor control, as letter are being traced. Experimental analyses also help identify specificities of eye writing, as compared to handwriting, and the resulting technical challenges.

## Introduction

**Figure 1:**
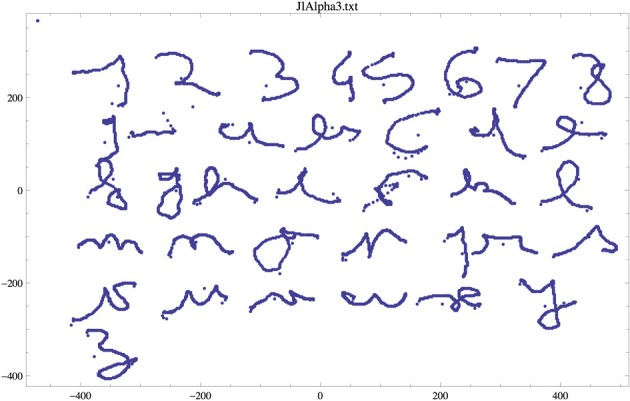
**Example of letters and digits written with his eyes by author JL (note that a spurious character, between the “9” and “a,” appears in this example, and was manually removed before further treatment)**.

The context of this paper is multi-disciplinary, as it concerns the computational study of writing in disabled patients. We use a novel apparatus, based on a particular static display and an illusory motion, which enables participants to generate smooth-pursuit movement at will and in the direction of their choice, without any external target. Coupled with an eye-tracking device, the system allows participants to “write” cursive letters with their eyes (Lorenceau, 2012). Figure 1 shows an example alphabet written using the apparatus by author Jean Lorenceau.

The mere possibility to write with the eyes requires a way to overcome the inherent limitations encountered when attempting to voluntarily generate eye movements, smooth movements in particular. In everyday life, smooth pursuit eye movements serve to maintain the image of a moving target,—e.g., a flying bird, a moving car—on the fovea, where visual acuity is best. They are reputed impossible without a target to track and, indeed, any attempt to smoothly move the eyes against a static background results in a sequence of fast saccadic eye movements interrupted by fixations (Lisberger et al., 1987). Since smooth eye movements resemble cursive hand writing in many ways, pursuit eye movements appear best adapted to eye writing. A visual illusion, derived from the “reverse-phi” illusion first described by Anstis (1970), helps to overcome the above-mentioned limitations. This illusion occurs when a static display made of hundreds of disks distributed over a gray background change luminance over time. In this situation, the whole display appears to move in the same direction as the eyes, thus providing a visual moving substrate against which smooth pursuit can develop and, with training, be generated at will to write letters, digits, or words.

Cursive eye writing is, to the best of our knowledge, an original research topic. Therefore, tools need to be adapted to this situation. For instance, the task of character recognition is usually considered as an offline problem, which is to say that a completed, printed mark is assumed as input, from which letter identity has to be recovered (Rehman and Saba, 2012). The context of eye writing, however, naturally leads to consider the online variant of character recognition: the system records the user's eye movements, so that letters can be recognized as they are being written (Tappert et al., 1990; Wakahara et al., 1992; Plamondon and Srihari, 2000). Input is typically a matrix of pixels in the offline case, and an ordered sequence of *x, y* coordinates in the online case.

A careful study of the characteristics of trajectory produced by the eye, using this novel apparatus, is also required. For instance, the noise characteristics of eye movements and hand movements are different. In handwriting, involuntary pen-ups during letter tracing are seldom observed in adults, whereas their analog in eye writing that is to say, spurious saccades, have an unknown frequency and incidence on character recognition. Another open question concerns the impact of ocular microtremor (OMT) superposed to pursuit trajectories: it might make the drawing of smooth curves difficult, or might, on the other hand, easily be filtered from the input trajectory (Sheahan et al., 1993; Bolger et al., 1999; Martinez-Conde et al., 2004).

In this context, our objectives are two-fold. The first concerns adapting the Bayesian Action-Perception (BAP) model of reading and handwriting (Gilet et al., 2010, 2011) to the task of eye writing. We will call this new model of “eye reading” and “eye writing” the Bayesian Action-Perception for Eye On-Line model (BAP-EOL).

Our main goal is for the BAP-EOL model to show the feasibility of character recognition, in an online manner; that is to say, as the eye writes the character. Because the model internally represents letters using both relative position and velocity information, and because it is based on Bayesian inference, it is well-suited to this task; it provides and refines probabilistic estimates, as data evidence is accumulated, even in the case of missing and noisy information. Also, the BAP-EOL model is based on experimental learning and probability distributions, and is thus well-suited to deal with novelty detection that is to say, detect when a new symbol is to be added to the database of learned symbols.

The second objective is to adapt the BAP model to the specific context of writing in disabled patients. Many systems are already commercially available for this purpose. They usually consist in displays where common words, letters, and sentence fragments are presented visually; the user usually has to saccade to and fixate the desired item, and then blink to validate the selection. It is probably not the case that cursive eye-writing would compete, in terms of communication throughput, with commercially available solutions of this kind, using virtual keyboards and sophisticated word prediction. Therefore, at this stage of the project, we mostly focus on a proof-of-concept system that includes character recognition, as a scaffold to further explore alternative tasks specific to the context of cursive eye writing.

The first of these tasks is novelty detection. Indeed, contrary to these systems, eye writing based on smooth pursuit offers a means to communicate in a less constrained manner, since arbitrary trajectories can be drawn or written. Personality and creativity can be preserved, and conveyed in written messages, along with semantic content. Also, usual motor programs for letter tracing can be used while eye writing: this may bring ease of use and comfort to disabled patients. This is also a means toward code convergence: the user can add symbols to the vocabulary of known characters, which can be variants of usual motor programs (e.g., simplified letters), or new, arbitrary trajectories with semantic content (e.g., iconic drawings). These trajectories have to be automatically detected as new symbols by the system.

The second auxiliary task, specific to the context of eye writing, is disability assessment. In the BAP-EOL model, and assuming the eye writing apparatus is used by disabled patients, we describe the way the model can be used, as letters are being traced, to measure and track the evolution of motor characteristics in the produced movements. In other words, we consider eye writing as an opportunistic observation window into the fine motor control capabilities of disabled patients. To extend the model in this direction, we leverage the computational flexibility of probabilistic models: instead of providing a function, a probabilistic model encodes preliminary knowledge that can then be used in a variety of related tasks (Lebeltel et al., 2004; Colas et al., 2010; Diard et al., 2010). For instance, the BAP model was used to perform character recognition of course, but also writer recognition, letter production, character copy, and even forgery (i.e., copying letters using the writing style of another writer). Here, the BAP-EOL model includes a model of disability levels, and can thus, while recognizing characters, also assess the disability level of the user.

The rest of this paper is organized as follows. First, we present the BAP-EOL model, define formally the probabilistic model at its core, and show how Bayesian inference yields mathematical methods for letter recognition, novelty detection and disability assessment. We then present preliminary experimental results for each of these tasks, and discuss a roadmap for future developments.

## Materials and methods

### BAP-EOL model

The model is in two parts. The first part consists of a deterministic algorithm that analyzes trajectories recorded by the system. The second part is a probabilistic model that encodes knowledge about the way letters are traced, according to the symbol being traced and disability level of the user.

The first, algorithmic part of the model consists in extracting information from the input signal. Trajectories are recorded by the system in the form of a sequence of *x, y* positions, along with pupil diameter *d*, from initial time step 0 to current time *N*. As the trajectory is being traced, *x* and *y* velocities are computed by a finite difference approximation.

We summarize each trajectory as a sequence of via-points. Via-points are particular points where either the *x*-velocity or *y*-velocity is zeroed, or both [see Gilet et al. (2011) for a discussion of this choice of feature]. Additionally, starting points and end points of trajectories are via-points. Given a trajectory, we only memorize via-points, where we record both position and velocity information. Because there is no reference frame for writing when using the eye writing apparatus (the user can write in any portion of the screen displaying the illusion), absolute positions are not meaningful. Instead, we record relative displacements along the trajectory: the first via-point, which corresponds to the beginning of the trajectory, is associated with position *(0, 0)*. Via-points other than the first are associated with *C*^*k*^_Δ*x*_, *C*^*k*^_Δ*y*_, the relative *x* and *y* displacements performed since the preceding via-point. At each via-point, we also record, *C*^*k*^_*ẋ*_ and *C*^*k*^_*ẏ*_, the *x* and *y* velocities at the *k*-th via-point.

**Figure 2:**
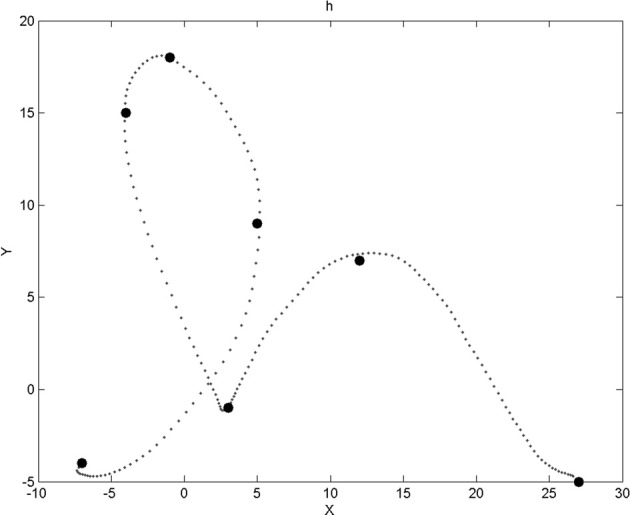
**Example of a trajectory for the letter “h” and the corresponding via-points positions.** Via-points, where *x* and *y* velocities are zeroed, perceptually corresponds to extremes portions of the trajectories (i.e., bottom, top, left, and right borders of the local curvature). Notice that, after the first point of the trajectory, a via-point appears to be missing: it was filtered out by a geometric constraint that ensures that via-points are not too close to each other. Notice also that via-points sometimes appear out of the trajectory (and also in Figures 6, 7, 10, 14): this is because we visualize here via-point positions in the low precision, discrete domains of the corresponding probabilistic variables (see the experimental Results section).

A via-point is therefore four-dimensional, with two dimensions for relative position information and two dimensions for velocity information. For each trajectory, we memorize at most 15 via-points (which is more than enough, in practice). Figure 2 shows an example trajectory and the corresponding via-points.

When a trajectory is finished (either because a completed letter was recognized or the user blinked to explicitly start the next trajectory), its width *S*_*x*_ and height *S*_*y*_ are computed. To quantify the effect of high-frequency components in eye movements during trajectory production (undetected spurious saccades, OMT), we compute the Fourier Transform of the velocities of the trajectory. From the resulting frequency spectrum, we compute the overall spectral energy above some frequency threshold *A* = ∫^∞^_*f*_0__|*X*(*f*)|^2^ *df*, where *X* represents the Fourier Transform. In our preliminary experiments, and after inspection of typical frequency spectra, we set the threshold at *f*_0_ = 2.6 Hz so to separate smooth-pursuit movements from other components.

Finally, we record the mean μ_*p*_ and variance σ_*p*_ of pupil diameters recorded during trajectory production. Pupil diameter is mainly influenced by light exposure, emotional state, and attention level of the subject: these are assumed to be constant during the production of isolated letters. In most eye-tracking system, algorithms used for pupil detection affect measurement of eye direction; errors due to this effect are cancelled out in our case, since we only compute relative displacements. In other words, pupil diameter mean sizes and variations will be assumed to be independent of geometric information of traced trajectories, and thus assumed to be irrelevant for character recognition and novelty detection. They will, however, be useful for characterizing and recognizing disability levels. Overall, the set of auxiliary variables *S*_*x*_, *S*_*y*_, *A*, μ_*p*_, σ_*p*_ provide additional information to the local geometry of the trajectory, represented by via-points variables.

This completes the description of the first part of the model: it is implemented using a series of straightforward scripts. We do not provide more details in this limited space.

The second part of the BAP-EOL model is probabilistic, and includes two high-level variables of interest. Firstly, variable *L* encodes the vocabulary of known symbols. It is an unordered set of discrete values, *L* = {*a*, *b*, *c*, …, *z*, 1, 2, …, 9} (so far, the database we treat does not include the digit 0). Note that the symbols are labeled with letter alphabet and digits here to better illustrate and make the example more concrete; in practice, the system could handle and add to its vocabulary arbitrary symbols, including punctuation marks, foreign alphabets, or even smiley faces and geometric shapes, as long as the associated trajectories are geometrically reliably distinguishable from other symbols. Secondly, the disability level *H* is encoded into a 3-level discrete variable: *H* = {1, 2, 3}. Until we introduce disability level assessment, for simplicity, we will consider the case [*H* = 1, i.e., a healthy subject.

We define a joint probability distribution over previous variables, except the *x, y, d* input which is already pre-treated in the first part of the BAP-EOL model. In other words, we define *P*(*C*^1:15^_Δ*x*_, *C*^1:15^_Δ*y*_, *C*^1:15^_*ẋ*_, *C*^1:15^_*ẏ*_, *S*_*x*_, *S*_*y*_, *A*, μ_*p*_, σ_*p*_, *L*, *H*), with *C*^1:15^_Δ*x*_ as a shorthand for the sequence of variable *C*^*k*^_Δ*x*_ between index 1 and 15, i.e., *C*^1:15^_Δ*x*_ = *C*^1^_Δ*x*_, *C*^2^_Δ*x*_, …, *C*^15^_Δ*x*_. This probabilistic model is therefore 67-dimensional. To manage this dimensionality, we decompose the joint probability distribution into a product of low-dimensional terms, thanks to conditional independence hypotheses:

$$\begin{array}{l} P\text{​}\left(C_{\Delta x}^{1:15},C_{\Delta y}^{1:15},C_{\dot{x}}^{1:15},C_{\dot{y}}^{1:15},S_{x},S_{y},A,\mu_{p},\sigma_{p},L,H\right)\;=P\text{​}\left(L\right)\times P\text{​}\left(H\right)\;\times \;P\text{​}\left(C_{\Delta x}^{1:15}|L,H\right)\times P\text{​}\left(C_{\Delta y}^{1:15}|L,H\right)\;\times \;P\text{​}\left(C_{\dot{x}}^{1:15}|L,H\right)\times P\text{​}\left(C_{\dot{y}}^{1:15}|L,H\right)\;\times \;P\text{​}\left(S_{x}|L,H\right)\times P\text{​}\left(S_{y}|L,H\right)\times P\left(A|L,H\right)\;\times \;P\text{​}\left(\mu_{p}|H\right)\times P\text{​}\left(\sigma_{p}|H\right). \end{array}$$

We now define each term of this product. The first, *P* (*L*), is a prior probability distribution over letters, i.e., it encodes the likelihood of each letter before it is traced. In the preliminary experiments we describe below, we only consider isolated cursive letters so that no linguistic context and information is available. We thus define *P* (*L*) as a uniform probability distribution. Obvious improvements would include replacing this by a distribution reflecting frequency of letters in the user's language (e.g., the probability that an English speaking user would write an “e” would be high), or even informing this by a top-down process driven by word recognition and word completion (e.g., if the four previous letters were “tabl,” the probability that the next letter is an “e” would be high).

The second term, *P*(*H*), is a prior probability distribution over the disability level of the user, which we set as a uniform probability distribution.

The next four terms are each 15-dimensional, and of the form *P*(*C*^1:15^_Δ*x*_ |*L, H*). They are themselves decomposed into products of simpler terms (with similar equations for the *y*, **ẋ**, and **ẏ** dimensions):

$$P\text{​}\left(C_{\Delta x}^{1:15}|L,H\right)=P\text{​}\left(C_{\Delta x}^{1}|L,H\right)\prod_{i=2}^{15}P\text{​}\left(C_{\Delta x}^{i}|C_{\Delta x}^{i\;-\;1},L,H\right).$$

In other words, we record, for each letter and each disability level, a series of probability distributions over the relative positions and velocities at each via-point. Each of these distributions is a Laplace succession law, i.e., a variant of a frequency histogram where no value has 0 probability, even when unobserved (Murphy, 2006). The four probability distributions over via-points relative positions and velocities constitute a “probabilistic database:” the model encodes and memorizes probabilistic descriptions of geometry and dynamics of trajectories, and their association with symbols. It can also be understood as a variation upon classical HMM models of online letter recognition (Hu et al., 1996; Artières et al., 2007).

In a similar manner, the terms *P*(*S*_*x*_ |*L, H*), *P*(*S*_*y*_ |*L, H*), and *P*(*A* |*L, H*) memorize, for each letter and each disability level, probabilities about the widths, heights, and amplitude of high frequency components of the trajectory. Each is a truncated and discrete normal probability distribution, so that probabilities of negatives sizes and negative amplitudes are zero. Finally, the terms *P*(μ_*p*_ |*H*) and *P*(σ_*p*_ |*H*) are also defined by truncated, discrete normal probability distributions, but note that they are assumed to be independent of the letter *L*, given the disability level *H*. This completes the structural definition of the probabilistic model *P*(*C*^1:15^_Δ*x*_, *C*^1:15^_Δ*y*_, *C*^1:15^_*ẋ*_, *C*^1:15^_*ẏ*_, *S*_*x*_, *S*_*y*_, *A*, μ_*p*_, σ_*p*_, *L, H*).

### Parameter identification

The probabilistic model being structurally defined, we now describe how its parameters are learned. The learning scenario we consider here is a supervised method: we assume, as input, a database of trajectories that are labeled. In other words, each entry is a trajectory associated with the letter *L* it corresponds to, the disability level *H* of the user that produced it, and the identity of the user.

The deterministic scripts are applied on the trajectories, to compute values for the via-point variables *C*^1:15^_Δ*x*_, *C*^1:15^_Δ*y*_, *C*^1:15^_*ẋ*_, *C*^1:15^_*ẏ*_ and auxiliary variables *S*_*x*_, *S*_*y*_, A, μ_*p*_, and σ_*p*_. Therefore, all variables of the model have associated values and the learning process is straightforward. For instance, concerning terms of the form *P*(*C*^*i*^_Δ*x*_ |*C*^*i* − 1^_Δ*x*_, *L, H*), which are Laplace succession laws, occurrence counting is performed. Denoting *n*_*i*_ the number of times that value *c*_*x*_ has been observed for the *i*-th via-point in the trajectory database, we have:

$$P\text{​}\left(\left[C_{\Delta x}^{i}=c_{\Delta x}\right]|C_{\Delta x}^{i\;-\;1},L,H\right)=\frac{n_{i}+\alpha }{N+\alpha K},$$

where *N* is the total number of observations, *K* the number of possible cases for variable *C*^*i*^_Δ*x*_, and α a tuning parameter that controls learning speed.

A technical issue concerns the probability distributions for the *i*-th via-point learned from a trajectory that contains less than *i* via-points. Since we do not explicitly represent trajectory lengths with a probabilistic variable, we use instead special values, where we concentrate the probability mass. These special values are “outside” of the domains of *C*^*i*^_Δ*x*_, *C*^*i*^_Δ*y*_, *C*^*i*^_*ẋ*_, and *C*^*i*^_*ẏ*_. In other words, they act as termination markers, and are used as a “well” of probabilities. The via-points of already terminated sequences will be associated with very high probabilities of these special values that are geometrically meaningless.

Other probability distributions about via-points are also associated with Laplace succession laws. The remaining terms over auxiliary variables are associated with discrete probability distributions, which approximate Normal distributions: their means and standard deviations are computed in the usual manner.

### Using the model for online character recognition

Having defined both model components and learned the parameters of the probabilistic part, we turn to showing how it is used to solve tasks. The first is the task of online character recognition: as a trajectory is being traced, the algorithmic portion of the model will detect and provide via-point information as they occur. When the trajectory is finished, it also outputs information about the auxiliary variables (width, height, etc.).

The learned BAP-EOL probabilistic model can be used to compute, as via-points are detected, probability distributions over letters. In other words, the initial knowledge about letters is the prior probability distributions *P*(*L*), which is uniform. When the first via-point is detected, we compute *P*(*L* |*C*^1^_Δ*x*_, *C*^1^_Δ*y*_, *C*^1^_*ẋ*_, *C*^1^_*ẏ*_), when the second via-point is detected we compute *P*(*L* |*C*^1:2^_Δ*x*_, *C*^1:2^_Δ*y*_, *C*^1:2^_*ẋ*_, *C*^1:2^_*ẏ*_), etc. Finally, when the trajectory is completed and auxiliary variables become available, we compute *P*(*L* |*C*^1:15^_Δ*x*_, *C*^1:15^_Δ*y*_, *C*^1:15^_*ẋ*_, *C*^1:15^_*ẏ*_, *S*_*x*_, *S*_*y*_, *A*, μ_*p*_, σ_*p*_).

The BAP-EOL model being given, Bayesian inference dictates how these probability distributions are computed. We first show the symbolic probabilistic inference for the general case when the *i*-th via-point has been detected:

$$P\text{​}\left(L|C_{\Delta x}^{1:k},C_{\Delta y}^{1:k},C_{\dot{x}}^{1:k},C_{\dot{y}}^{1:k}\right)\;\propto \sum_{h\in H}\left(\begin{array}{l} P\text{​}\left(C_{\Delta x}^{1}|L,H\right)\times P\text{​}\left(C_{\Delta y}^{1}|L,H\right)\\ \times P\text{​}\left(C_{\dot{x}}^{1}|L,H\right)\times P\text{​}\left(C_{\dot{y}}^{1}|L,H\right)\\ \;\times \prod_{i=2}^{k}(P\text{​}\left(C_{\Delta x}^{i}|C_{\Delta x}^{i-1},L,H\right)\\ \;\times P\text{​}\left(C_{\Delta y}^{i}|C_{\Delta y}^{i-1},L,H\right)\\ \;\times P\text{​}\left(C_{\dot{x}}^{i}|C_{\dot{x}}^{i-1},L,H\right)\;\\ \times P\text{​}\left(C_{\dot{y}}^{i}|C_{\dot{y}}^{i-1},L,H\right)) \end{array}\right)$$

Prior probability distributions about letters *P*(*L*) and disability level *P*(*H*) have disappeared from the summation because they are assumed to be uniform probability distributions, and thus constant values, which can be incorporated into the normalization constant.

The Bayesian inference for the final term is:

$$P\text{​}\left(L|C_{\Delta x}^{1:15},C_{\Delta y}^{1:15},C_{\dot{x}}^{1:15},C_{\dot{y}}^{1:15},S_{x},S_{y},A,\mu_{p},\sigma_{p}\right)\;\propto \sum_{h\in H}\left(\begin{array}{l} P\text{​}\left(C_{\Delta x}^{1}|L,H\right)\times P\text{​}\left(C_{\Delta y}^{1}|L,H\right)\\ \;\times P\text{​}\left(C_{\dot{x}}^{1}|L,H\right)\times P\text{​}\left(C_{\dot{y}}^{1}|L,H\right)\\ \;\times \prod_{i=2}^{15}(P\text{​}\left(C_{\Delta x}^{i}|C_{\Delta x}^{i-1},L,H\right)\times P\text{​}\left(C_{\Delta y}^{i}|C_{\Delta y}^{i-1},L,H\right)\\ \;\times P\text{​}\left(C_{\dot{x}}^{i}|C_{\dot{x}}^{i-1},L,H\right)\times P\text{​}\left(C_{\dot{y}}^{i}|C_{\dot{y}}^{i-1},L,H\right))\\ \;\times P\text{​}\left(S_{x}|L,H\right)\times P\text{​}\left(S_{y}|L,H\right)\times P\text{​}\left(A|L,H\right) \end{array}\right)$$

Terms about pupil diameter size and variations vanish from the equations because they are assumed to be independent from letter identity, and thus can factored outside of the summation, where they sum to 1 by the normalization rule. *P*(*L* |*C*^1:15^_Δ*x*_, *C*^1:15^_Δ*y*_, *C*^1:15^_*ẋ*_, *C*^1:15^_*ẏ*_, *S*_*x*_, *S*_*y*_, *A*, μ_*p*_, σ_*p*_) is thus equivalent to *P*(*L* |*C*^1:15^_Δ*x*_, *C*^1:15^_Δ*y*_, *C*^1:15^_*ẋ*_, *C*^1:15^_*ẏ*_, *S*_*x*_, *S*_*y*_, *A*).

As via-points are accumulated, the probability distribution over letters is updated. When the end of the trajectory is reached, and auxiliary variables become available, the final probability distribution over letters is computed. From this distribution, the system selects the most probable letter as the recognized letter, and outputs its value.

### Using the model for novelty detection

The second task we consider is an extension of the previous one: we want the system to be able to detect when an input trajectory does not correspond to a known letter. To perform this task, we define a modification of the model, and a modification of the online character recognition process we just described (it was not introduced straightaway, for didactic purposes).

The symbol set of variable *L* is augmented with a special symbol, noted “$,” which represents the “unknown letter.” In the probabilistic model, many terms have variable *L* appearing on their right-hand side (i.e., after the conditioning solidus). Consider for instance, *P*(*C*^*i*^_Δ*x*_ |*C*^*i* − 1^_Δ*x*_,[*L* = *l*], *H*), the probability distribution over the relative *x*-position of the *i*-th via-point for any letter denoted *l*. When [*l* ≠ $], this probability distribution is the same as described above. On the other hand, when [*l* = $], this probability distribution is a uniform distribution. All other probabilistic terms conditioned on variable *L* are modified in the same manner: they are unmodified for known symbols, and are associated with uniform distributions for the symbol “$.”

The character recognition task with novelty detection is solved by a small variation of the previous algorithm: as before, we compute probability distributions over letters as via-points are detected but the case might happen that the unknown character “$” would the most probable at the end of the trajectory. In other words, we track, by Bayesian inference, the evolution of *P*([*L* = “$”]|*C*^1:15^_Δ*x*_, *C*^1:15^_Δ*y*_, *C*^1:15^_*ẋ*_, *C*^1:15^_*ẏ*_) as via-points are accumulated: the fact that auxiliary variables are not taken into account in this case will be justified experimentally below.

In the event that the unknown character “$” is the most probable, we propose to the user to create a new symbol value in the vocabulary set *L*, and associate the last trajectory to this new symbol to learn. This helps alleviate the previous assumption that learning is performed in a fully supervised manner: instead, the user traces a letter without providing a label. The system either recognizes it as a known character, stores the last trajectory into the database for this character, thus refining the learned probabilistic model, or the system detects that it is probably a new character, and asks the user for confirmation that it should create a new case in the *L* domain.

### Using the model for disability assessment

The third and final task we describe here concerns disability assessment, which is solved by computing probability distributions over disability level *H* instead of letter *L*. When a trajectory is completed, Bayesian inference yields:

$$P\text{​}\left(H|C_{\Delta x}^{1:15},C_{\Delta y}^{1:15},C_{\dot{x}}^{1:15},C_{\dot{y}}^{1:15},S_{x},S_{y},A,\mu_{p},\sigma_{p}\right)\;\propto \sum_{l\in L}\left(\begin{array}{l} P\text{​}\left(C_{\Delta x}^{1}|L,H\right)\times P\text{​}\left(C_{\Delta y}^{1}|L,H\right)\\ \;\times P\text{​}\left(C_{\dot{x}}^{1}|L,H\right)\times P\text{​}\left(C_{\dot{y}}^{1}|L,H\right)\\ \;\times \prod_{i=2}^{15}(P\text{​}\left(C_{\Delta x}^{i}|C_{\Delta x}^{i-1},\text{​}L,H\right)\times P\text{​}\left(C_{\Delta y}^{i}|C_{\Delta y}^{i-1},\text{​}L,H\right)\\ \;\times P\text{​}\left(C_{\dot{x}}^{i}|C_{\dot{x}}^{i-1},\text{​}L,H\right)\times P\text{​}\left(C_{\dot{y}}^{i}|C_{\dot{y}}^{i-1},\text{​}L,H\right))\\ \;\times P\text{​}\left(S_{x}|L,H\right)\times P\text{​}\left(S_{y}|L,H\right)\times P\text{​}\left(A|L,H\right)\\ \;\times P\text{​}\left(\mu_{p}|H\right)\times P\text{​}\left(\sigma_{p}|H\right) \end{array}\right)$$

When a sequence of trajectories is recorded, the average probability distribution over disability level can be computed and recorded, for later inspection by the medical staff (i.e., there is no claim to provide automatic diagnostic, merely a measurement tool).

## Results

We report here a series of experimental results based on a preliminary database consisting of the first ever alphabets produced using the eye-writing apparatus. Author Jean Lorenceau wrote 9 alphabets, with a few missing characters (some “z,” for instance), and 1–9 digits (but no 0). One of these is shown Figure 1.

### Data filtering and deterministic variable extraction

In these initial experiments, data was collected at 75 Hz. Trajectory positions and velocities have both been filtered by a binomial filter of order 20, in order to smooth trajectories and remove noise in the data acquisition process.

When the user starts a new trajectory, a saccade is commonly performed first to fixate the eye near some usual starting position on the screen. Then movement is initiated, but this process is, contrary to handwriting, not immediate. Some time is needed to perceive the illusion and enter smooth-pursuit with voluntary control: the eye first has to move a bit so as to receive stimulation interpreted as movement in the same direction, which reinforces the perception of an illusory moving target, which makes movement in the same direction easier, so that the eye moves a bit more in the same direction, etc. This “burn-in” period is highly susceptible to containing intrusive saccades of short amplitude, or meandering, weakly directed movements. End portions of trajectories also can be contaminated in this manner, with small amplitude saccades preceding a blink usually associated with trajectory termination.

**Figure 3:**
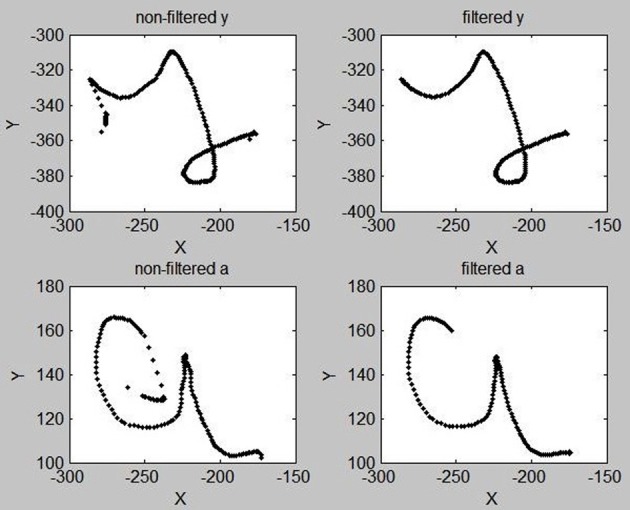
**Examples of “y” and “a” letters before (Left) and after (Right) the trimming process designed to remove intrusive saccades at the beginnings and ends of trajectories, and the filtering of high frequency components**.

Overall, most of these intrusive saccades are easily filtered out: we compute, for the first 30 and last 5 points of a trajectory, the acceleration. If the acceleration of the *i*-th point is greater than some threshold (empirically set to 0.4 space-unit/time_unit^2^), then we delete points 1 to *i* of the trajectory. In most cases, this successfully removes intrusive saccades related to the movement initiation and movement release periods of the beginning and end of trajectories. Figure 3 shows two examples of this trajectory trimming process.

**Figure 4:**
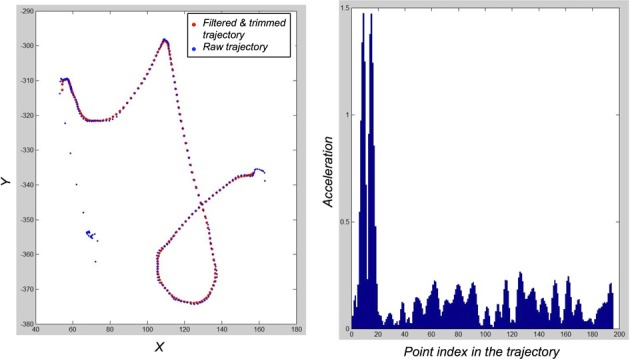
**Illustration of the filtering and trimming of trajectories. Left:** Raw trajectory for a “y” exemplar (blue dots), and the resulting filtered and trimmed trajectory (red dots). **Right**: acceleration profile of the raw trajectory. Spurious saccades in movement initiation correspond to easily detected and filtered peaks.

This trajectory trimming process is robust with respect to the chosen threshold value for acceleration. Figure 4 shows a typical acceleration profile for a raw trajectory in the database: saccades correspond to large peaks in acceleration amplitude. Any threshold value between 0.3 and 1 space-unit/time_unit^2^would roughly yield identical trimmed trajectories from the raw trajectories in our database. This trimming process is, however, slightly sensitive to the number of points that can be trimmed at the beginnings and ends of trajectories: the trade-off between data clean-up and conservation of information was set empirically once, achieving satisfactory compromise, without further parameter fiddling.

In some cases however, some saccades and meandering movements remain in the trajectories processed in our experiments. The impact of these superfluous movements is larger at the beginning of trajectories, because they induce extraneous via-points, and our probabilistic model is sensitive to via-point insertion. Notice that, in this preliminary version, the BAP-EOL model does not include an explicit model of via-point insertion and deletion. This has a negative impact on the recognition rates reported here, and warrants further consideration.

We process the filtered and trimmed trajectories to extract a maximum of 15 via-points on each. To prevent via-points to being too close from one another, we have set a geometric constraint: the *x* and *y* distances between two successive via-points have to be greater than a small threshold. This successfully removes superfluous via-points along vertical and horizontal plateaus, for instance. Figure 2 shows an example of via-points detected for an instance of the letter ‘h’: a via-point right after the start of the trajectory was removed by this geometric criterion.

The auxiliary variables are also computed at this stage. Overall, from each trajectory, after the deterministic scripts, we obtain and memorize 67 parameters: 15 4-dimensional via-points, 5 auxiliary variables, the letter identity *L*, and disability level *H*.

### Parameters of the probability distributions in the BAP-EOL model

To compute parameters of the probability distributions in the BAP-EOL model, we used a cross-validation method (Russell and Norvig, 1995; Shiffrin et al., 2008): out of the 9 available alphabets, we learn the parameters on 8 alphabets and use the ninth for testing purposes. For instance, the letter identification task is performed on each character in this ninth alphabet. The procedure is repeated 9 times, each alphabet serving once for testing, and 8 times for learning. Except for illustrative examples, we present below average results during this whole process.

We first instantiate the probabilistic variables of the BAP-EOL model with the following discrete domains:

- via-points, *C*^*k*^_Δ*x*_ and *C*^*k*^_Δ*y*_, each have 81 possible integer values from −40 to 40, along with a special value for the probability “well” for via-points beyond trajectory termination;
- variables concerning velocities at via-points, *C*^*k*^_*ẋ*_ and *C*^*k*^_*ẏ*_, each have 21 possible integer values from −10 to 10 (scaling down by a factor 30 initial velocities measured in pixels/s), along with a special value for the probability “well” for via-points beyond trajectory termination;
- variables concerning width and height of completed trajectories, *S*_*x*_ and *S*_*y*_, each have 51 possible positive integer values, from 0 to 50;
- the variable concerning high-frequency components of trajectories, *A*, has 31 possible positive integer values, from 0 to 30;
- the variable concerning the average pupil diameter, μ_*p*_, has 201 possible real values, from −1 to 1, with increments of 0.01, and a 0 corresponding to the pupil diameter during calibration of the apparatus;
- the variable concerning the variability of the pupil diameter, σ_*p*_, has 51 possible real values from 0 to 5, with increments of 0.1;
- the variable concerning the letter identity, *L*, has 36 possible symbolic values, for nine digits (1 to 9), 26 characters (“a” to “z”) and the unknown character (“$”);
- finally, the variable concerning the disability level, *H*, has 3 possible discrete degrees, 1 to 3.

Because the probabilistic model is completely discrete, the choice of domain sizes for these variables is directly related to computation time (having more values implies longer computations) and precision of representation (having less values implies more approximations). In our experiments, a satisfactory compromise was achieved directly, in this trade-off between numerical and time performance, without much empirical exploration.

We now turn to the parameters of the learned probability distributions in the BAP-EOL model. Terms about via-points relative positions and velocities are Laplace succession laws: their learning speed parameter α is set to a very small value (10^−7^), so that the initial uniform prior quickly vanishes. In the preliminary experiments we report here, and in order to counteract the small size of our learning database, we smooth the obtained Laplace probability distributions by a Gaussian filter: this makes the probability peaks “ooze” over neighboring values, simulating a larger database containing more trajectories, with some variability in the letter shapes. Gaussian filters are of order 15 and variance 2 for relative position terms, and order 7 and variance 1 for velocity terms.

**Figure 5:**
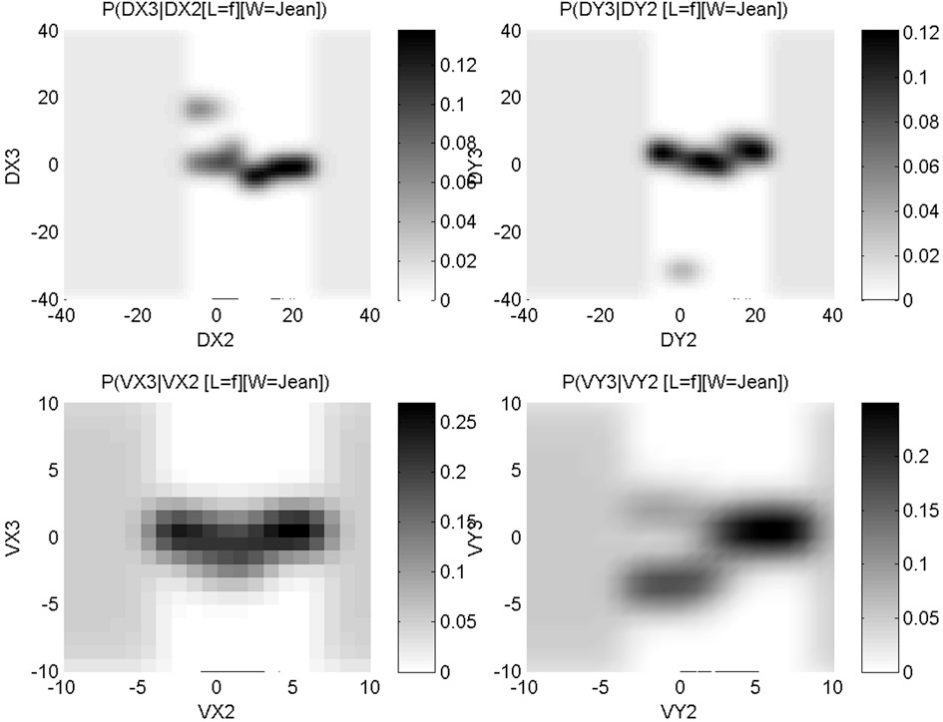
**Learned conditional probability distributions about *x* relative positions (top left panel), *y* relative positions (top right panel), *x* velocities (bottom left panel) and *y* velocities (bottom right panel), for the third via-point of letter “f.”** Each panel shows a *P(A*|*B)* conditional probability distribution, with the conditioning variable *B* on the *x*-axis, the domain of variable *A* on the *y*-axis; in other words, each column is a probability distribution that sums to unity. Probability values correspond to gray-levels: the darker the color, the higher the probability. See main text for interpretation of the information encoded in these probability distributions.

Figure 5 shows conditional probability distributions for the third via-point of the letter “f”: *P*(*C*^3^_Δ*x*_ |*C*^2^_Δ*x*_, [*L* = “f”], [*H* = 1]) (top left panel), *P*(*C*^3^_Δ*y*_ |*C*^2^_Δ*y*_, [*L* = “f”], [*H* = 1]) (top right panel), *P*(*C*^3^_*ẋ*_ |*C*^2^_*ẋ*_, [*L* = “f”], [*H* = 1]) (bottom left panel) and *P*(*C*^3^_*ẏ*_ |*C*^2^_*ẏ*_, [*L* = “f”], [*H* = 1]) (bottom right panel). It can be seen that most signal is concentrated on a geometrically common phenomenon that is, the third via-point is usually at the top of the loop of the “f,” and the eye is, at that moment, going leftward. Despite this commonality, there is variability in the learned trajectories: this variability is explicitly encoded in the probability distributions, with probability peaks that are close to each other. For instance, considering *y* relative displacements, most of the probability mass encodes an upward movement between the second and third via-point, given that there also was an upward movement previously, between the first and second via-point: this describes the upward motion for the first loop of an “f.”

**Figure 6:**
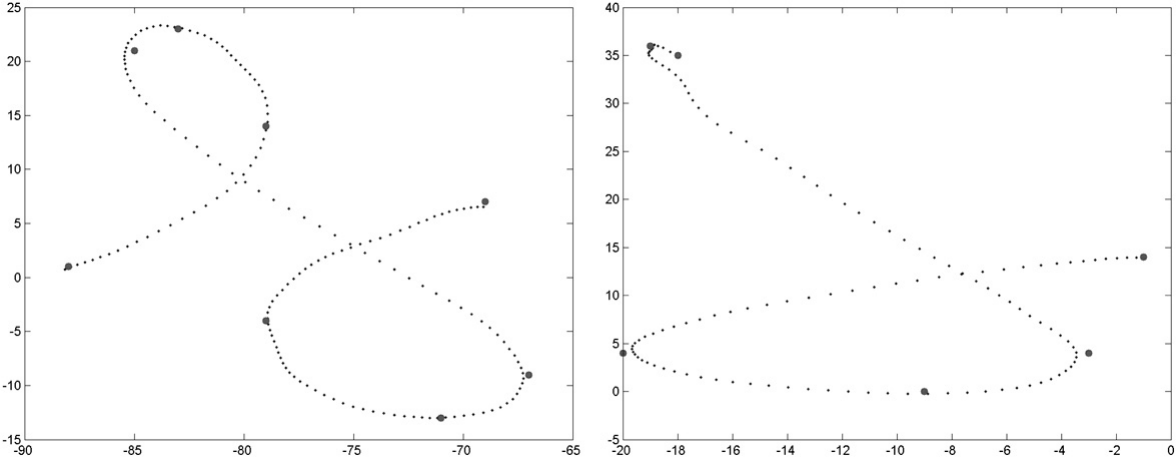
**Two allographs of the letter “f” present in the learning database. Left:** the most common geometric form, where the third via-point is at the top of the first loop. **Right**: the less common geometric form (present once in our database), where the first loop is reduced and the third point is instead at the beginning of the second loop, in the bottom portion of the trajectory.

However, some probability mass can clearly be seen to be discordant with this general shape. For instance, again considering *y* relative displacement, we observe a probability peak for a large downward movement, given a very small previous *y* displacement: the small gray patch around *P*([*C*^3^_Δ*y*_ = − 35] | [*C*^2^_Δ*y*_ = 0], [*L* = “f”], [*H* = 1]). This is due to the presence of another allograph in the learning database that is to say, an “f” exemplar with a different geometric shape. This is shown Figure 6. In our probabilistic model, such allographs are represented numerically in the probability distributions in this manner, and do not need an explicit encoding at the symbolic level (Gilet et al., 2011).

When parameters are fully identified from a database of trajectories, there are 21,829,107 computed parameters. The vast majority concerns the geometric information at via-points: for instance, a term *P*(*C*^*i*^_Δ*x*_ |*C*^*i* − 1^_Δ*x*_, *L, H*) requires 82 * 82 * 36 * 3 = 726,192 parameters. Of course, most of these parameters are not independent, either because of normalization constraints (probability distributions sum to unity over their domains), or because they are correlated by the filtering processes, or even thanks to underlying regularizing assumptions (e.g., some distributions are approximated Normal distributions). However, this still illustrates the scarcity of data in the preliminary experiments we report here: these parameters are learned from only 9 full alphabets, i.e., 35 * 9 = 315 characters. These parameters numerically fully define the joint probability distribution of the BAP-EOL model, *P*(*C*^1:15^_Δ*x*_, *C*^1:15^_Δ*y*_, *C*^1:15^_*ẋ*_, *C*^1:15^_*ẏ*_, *S*_*x*_, *S*_*y*_, *A*, μ_*p*_, σ_*p*_, *L, H*). It is now ready to be used for solving tasks.

### Online character recognition: experimental results

**Figure 7:**
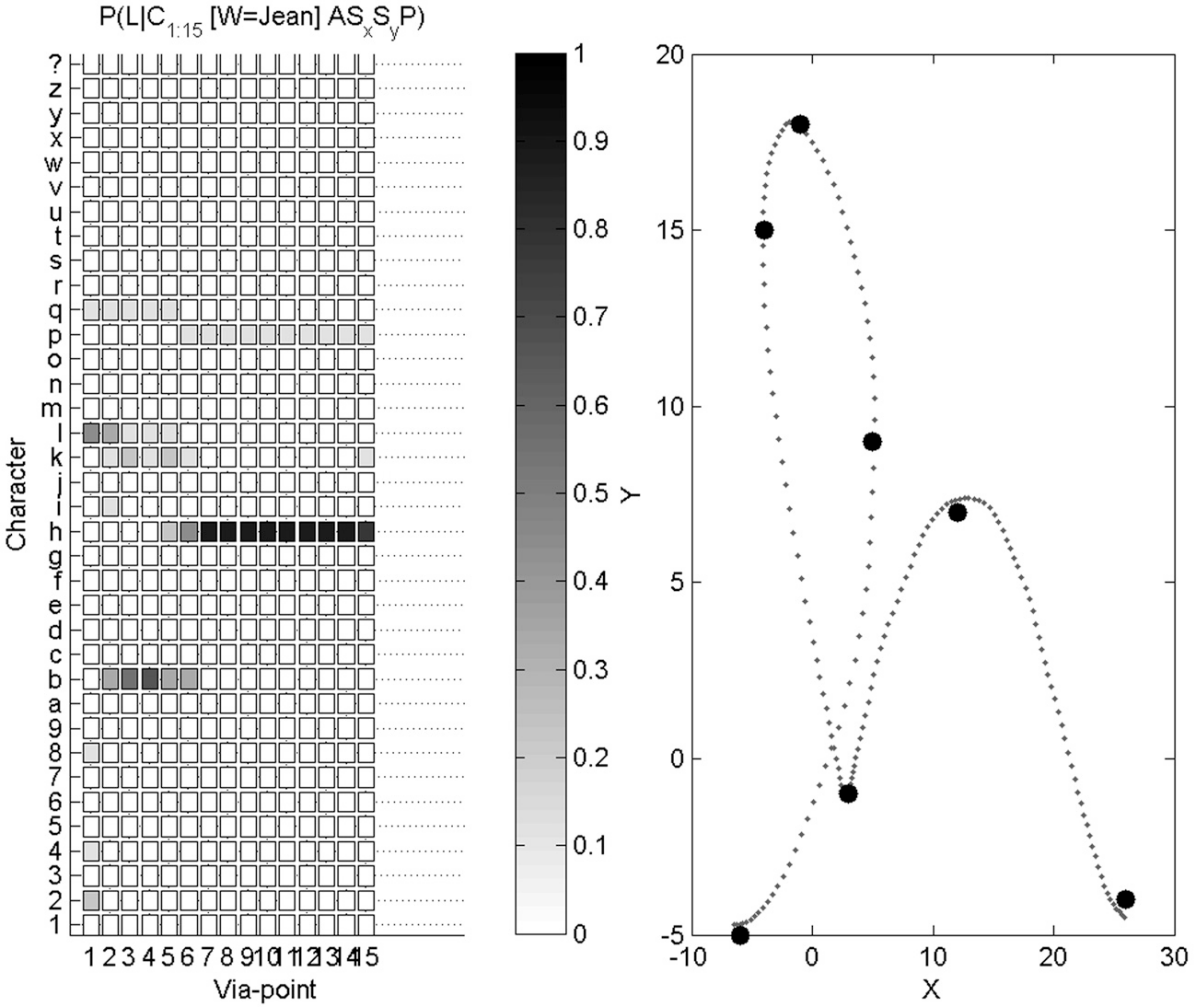
**Example of character recognition. Right:** the trajectory presented as input to the system, and the via-points detected along this trajectory. **Left**: probability distributions over letters as via-points are detected (the darker the color, the higher the probability): the first column is the probability distribution *P*(*L* |*C*^1^_Δ*x*_, *C*^1^_Δ*y*_, *C*^1^_*ẋ*_, *C*^1^_*ẏ*_), the second column is *P*(*L* |*C*^1:2^_Δ*x*_, *C*^1:2^_Δ*y*_, *C*^1:2^_*ẋ*_, *C*^1:2^_*ẏ*_), etc., and the final column is *P*(*L* |*C*^1:15^_Δ*x*_, *C*^1:15^_Δ*y*_, *C*^1:15^_*ẋ*_, *C*^1:15^_*ẏ*_, *S*_*x*_, *S*_*y*_, *A*).

The first task is character recognition. We compute, thanks to Bayesian inference, *P*(*L* |*C*^1:15^_Δ*x*_, *C*^1:15^_Δ*y*_, *C*^1:15^_*ẋ*_, *C*^1:15^_*ẏ*_, *S*_*x*_, *S*_*y*_, *A*). We introduce the experimental results with an illustrative example, shown Figure 7.

On this example, the letter to be recognized is an “h.” After the first via-point is detected, the probability distribution over letters is still close to a uniform distribution, and therefore highly uncertain. We recall that the only relevant information carried by the first via-point is velocity information, since position at the first via-point is (0, 0), whatever the letter. From the second via-point, the probability distribution gets concentrated around four peaks, related to the 4 letters “h,” “e,” “b,” and “l”: all of these, in cursive form, share a common geometric beginning, with an upward loop. From the sixth via-point however, the probabilities of letters other than “h” are very small. At the final stage, auxiliary variables are measured and taken into account. This example ends in a correct recognition, with a very high probability that the presented letter is an “h.”

**Figure 8:**
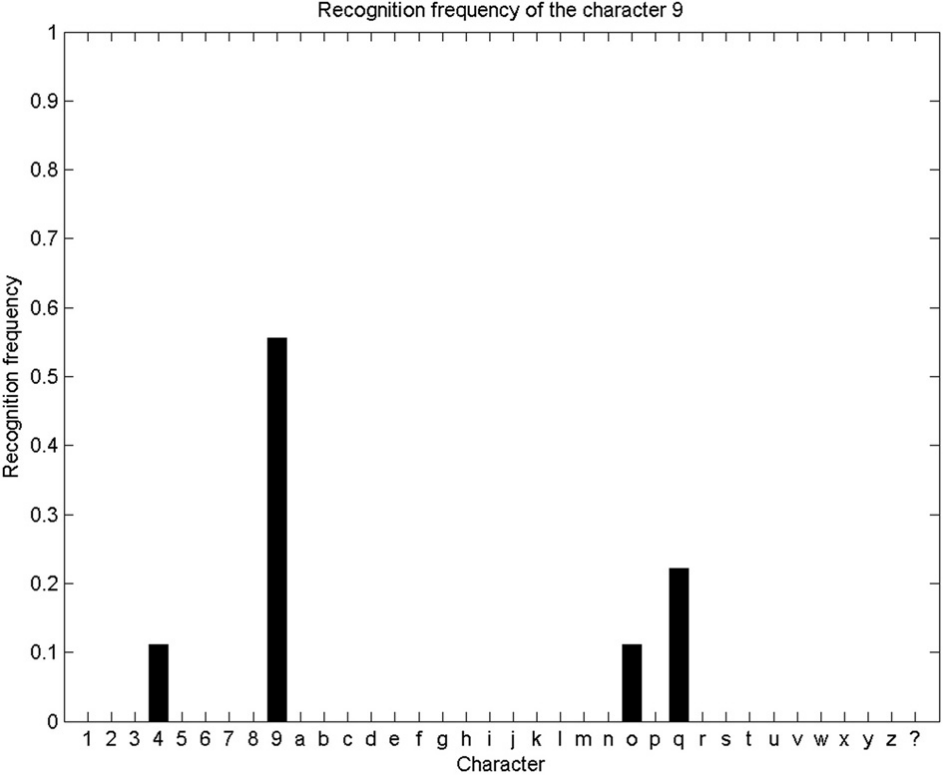
**Recognition frequency of the digit “9”: the system correctly recognizes the presented trajectory as a “9” five times, and incorrectly recognizes it as a “4” once, as a “o” once, and as a “q” twice.** Overall, this means a correct recognition rate of “9” of 55%.

We repeat this character recognition process over our whole database. For each trial, we compute the probability distribution over letters as described and illustrated above, and the system outputs the letter with the maximum final probability. Since our database contains 9 alphabets, each letter can be presented 9 times for recognition (and parameter learning is performed on the remaining 8 alphabets). We show Figure 8 the repartition of character recognition when digits “9” are presented.

**Figure 9:**
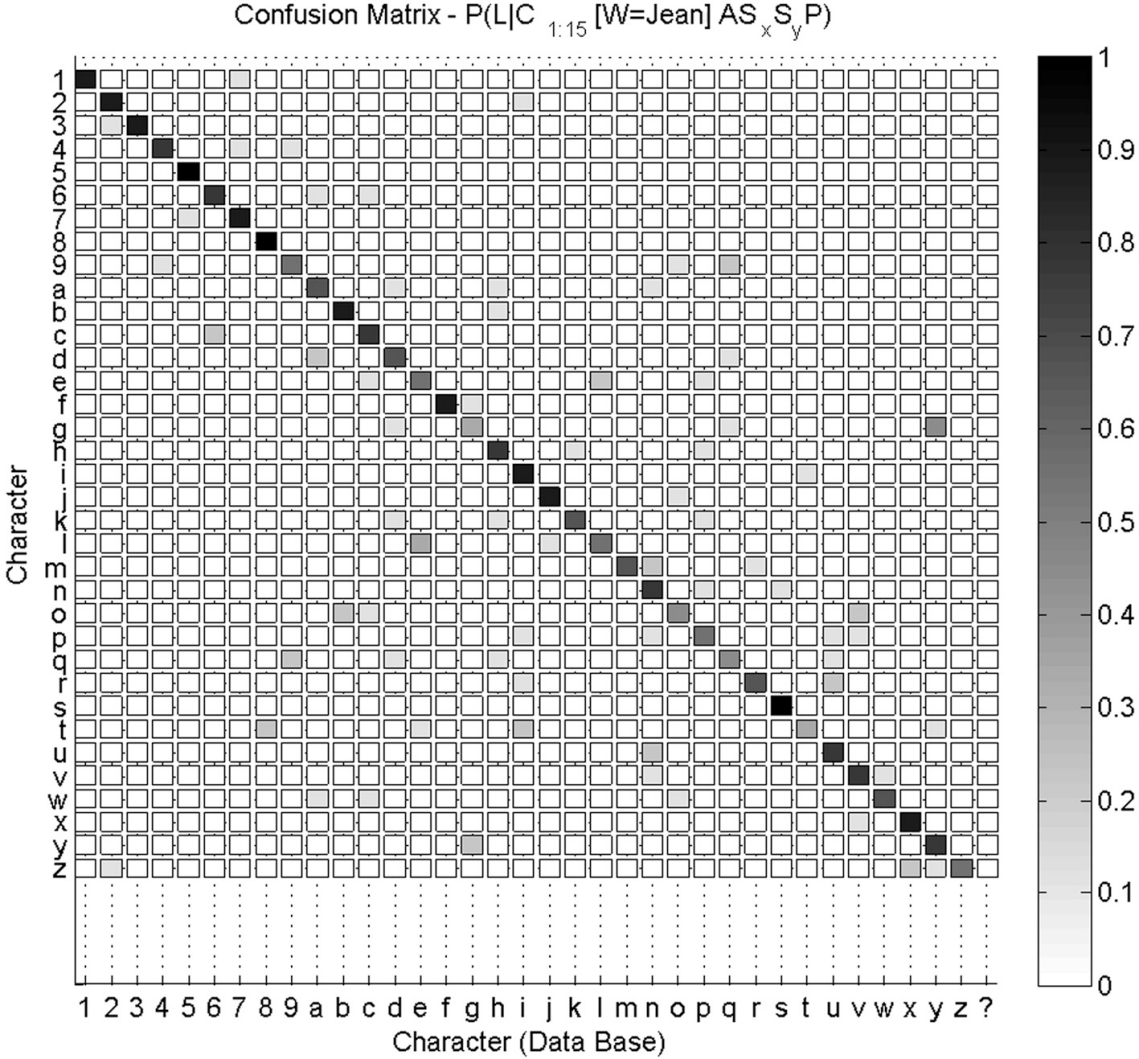
**Confusion matrix for character recognition.** Each row is a frequency count of characters recognized by the system (e.g., row labeled “9” was shown previously Figure 8), with darker colors associated with higher frequency counts. This confusion matrix is not square, as the 35 characters can also be recognized by the system as an unknown character (last column). Values on the diagonal indicate correct recognitions.

Overall, a 35 * 36 confusion matrix summarizes our experimental results for character recognition: for each of the 35 characters, they can either be recognized as one of the 35 known characters, or as an unknown character “$.” We show this confusion matrix Figure 9. We compute the global recognition rate of our experiment as the average fraction of correct recognitions for all letters (values on the diagonal of the confusion matrix). This global recognition rate is 68.6% (216 characters correctly recognized out of 35 * 9 = 315 tests).

**Figure 10:**
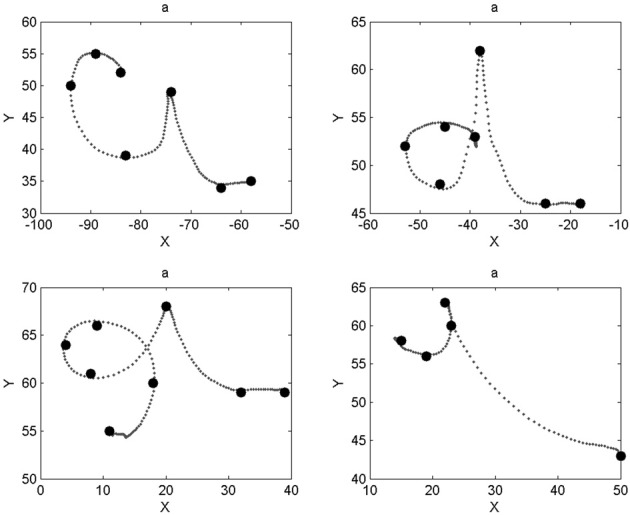
**Different letters “a” present in the database.** The two trajectories in the top row are correctly identified as letter “a” (the writer performs letter “d” differently than in the top right panel). The example in the bottom left panel is incorrectly recognized as a “b” (very similar shape except for a longer downward stroke during the first loop); the example in the bottom right panel is incorrectly recognized as a “t” (most “t” exemplars do not have a bar).

We notice that most characters are well-recognized, like digits “5” and “8” or letters “f” and “s.” Inspection of the database reveals that the writer had great regularity when drawing these characters, which makes their geometric probabilistic descriptions reliable. In some other cases, like letters “a,” “g,” “o,” or “t,” recognition is worse. Indeed, the eight learned trajectories already contain allographs or ill-formed trajectories, which make the learned model less stable. This is shown Figure 10 in the case of letter “a.”

**Figure 11:**
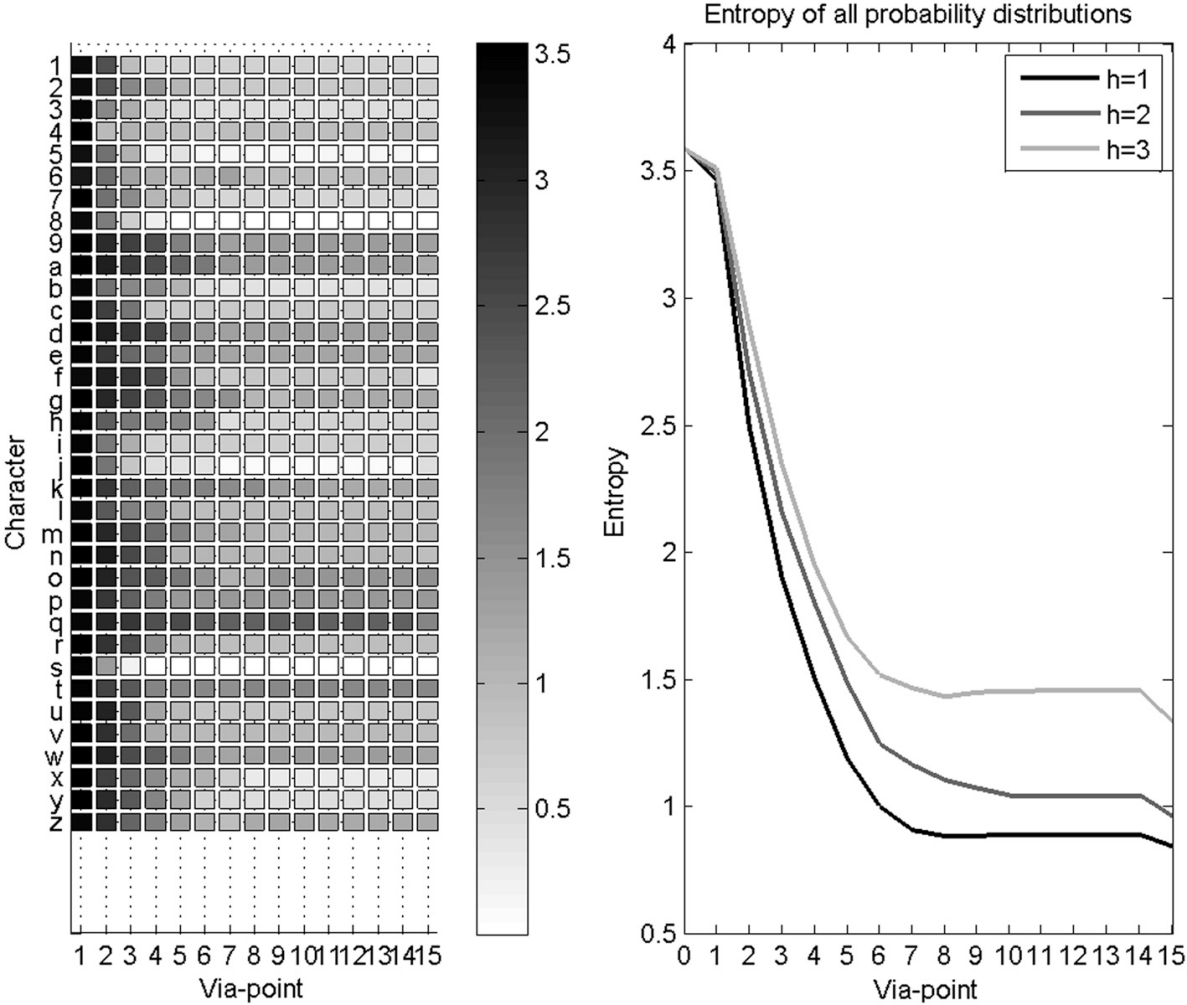
**Evolution of entropy during character recognition. Left panel:** each row is the mean Shannon entropy of the probability distribution over letters, as via-points are accumulated, for a given letter. **Right panel**: average entropy as via-points are accumulated, for all letters. Three curves are shown, one for each disability level: higher disability leads to slower convergence speed.

In some cases, characters are reliably recognized before their completion. Since we are interested in providing rapid and early character recognition (for instance, for word recognition and thus the prediction of upcoming letters), we study the convergence speed of the recognition process. We measure this convergence speed using the entropy of the probability distributions over letters as via-points are detected and accumulated. This is shown Figure 11. Recall that the Shannon entropy of a probability distribution *P* (*L*) represents its concentration, and is computed as:

$$H\text{​}\left(P\left(L\right)\right)=-\sum_{l\in L}P\text{​}\left(\left[L=l\right]\right)\log P\text{​}\left(\left[L=l\right]\right).$$

Before the first via-point is detected, the probability distribution over letters is uniform, with maximal entropy. The first via-point only brings initial velocity information, and thus the gain in entropy is less than for subsequent via-points. Finally, after the last via-point, auxiliary variables bring additional information, and entropy reaches its minimal value in this application.

We observe Figure 11 a fast convergence speed with a rapid decrease of entropy while the first 7 via-points are detected. Since most recorded trajectories are not much longer, the gain in information then reaches a plateau, until auxiliary variables become available after the fifteenth via-point. However, this hides some variability in recognition speed for different characters: some characters are recognized using only a few via-points (e.g., characters with short trajectories, or characters with characteristic beginnings, like the “s”), whereas uncertainty remains longer for other characters (e.g., “9” and “q” only differ at their end).

### Novelty detection: experimental results

The second task we solve with the BAP-EOL model is novelty detection that is to say, recognizing when the presented trajectory should be associated to a new symbol. In that case, the system asks for input and confirmation by the user.

**Figure 12:**
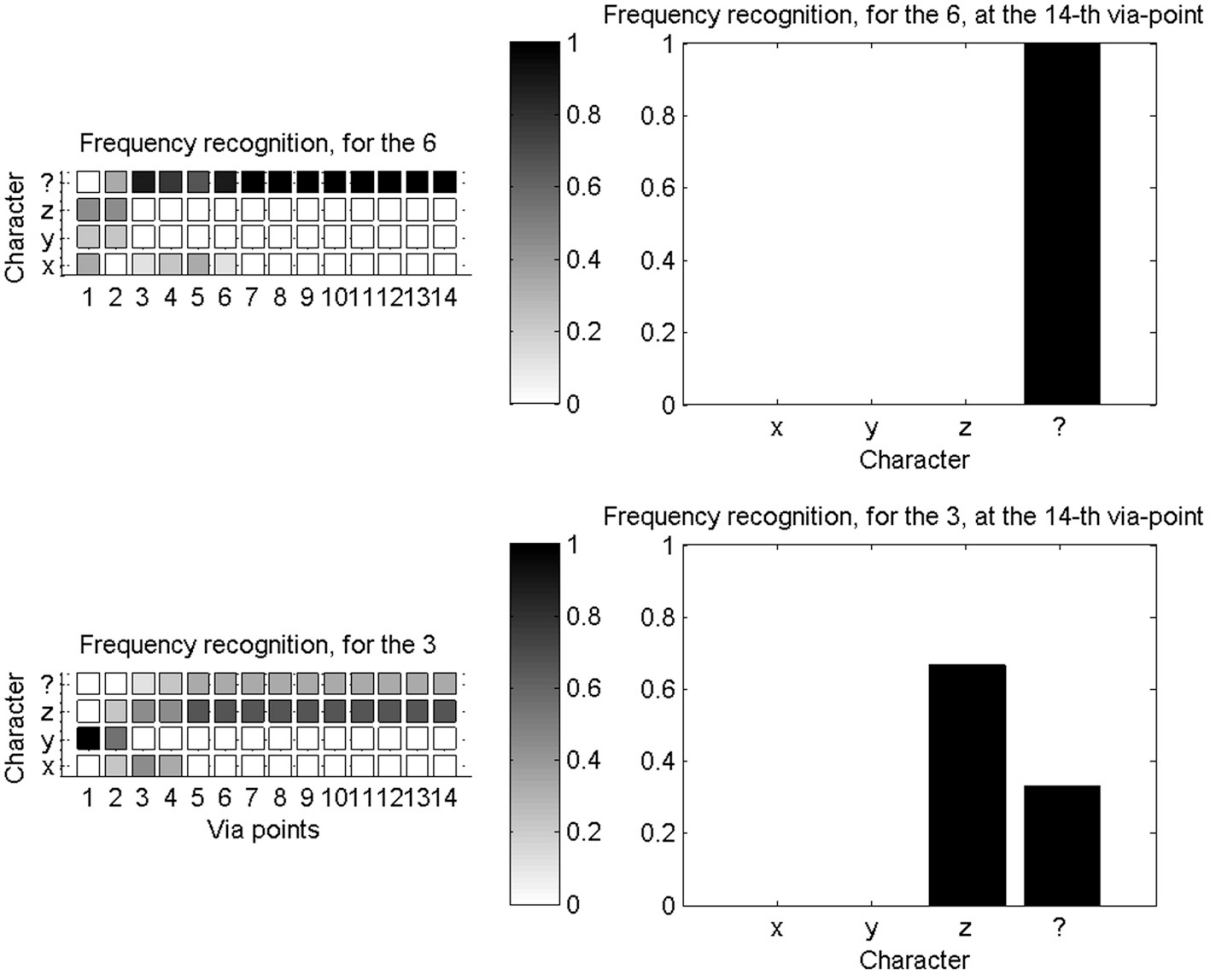
**Novelty detection during character recognition. Left column:** evolutions of the probability distributions over characters (among “x,” “y,” “z,” and “$”), as the fourteenth first via-points are detected. **Right column**: final probability distribution over letters after detection of the fourteenth via-point. Top row concerns recognition and novelty detection when a “6” is presented: it is outside of the learning database and correctly recognized as a new character. Bottom row is when a “3” is presented: while outside of the learning database, it is geometrically close to a “z,” and incorrectly recognized as such, most of the time.

To experimentally test novelty recognition, we reduce our learning database to a subset of available characters: we only learn parameters for the “x,” “y,” and “z” characters. We then proceed with character recognition, and the system can only either recognize one of the three known characters, or the unknown character “$.” Figure 12 shows two illustrative examples of probability distributions for this small set of recognizable character, as the first 14 via-points are detected.

**Figure 13:**
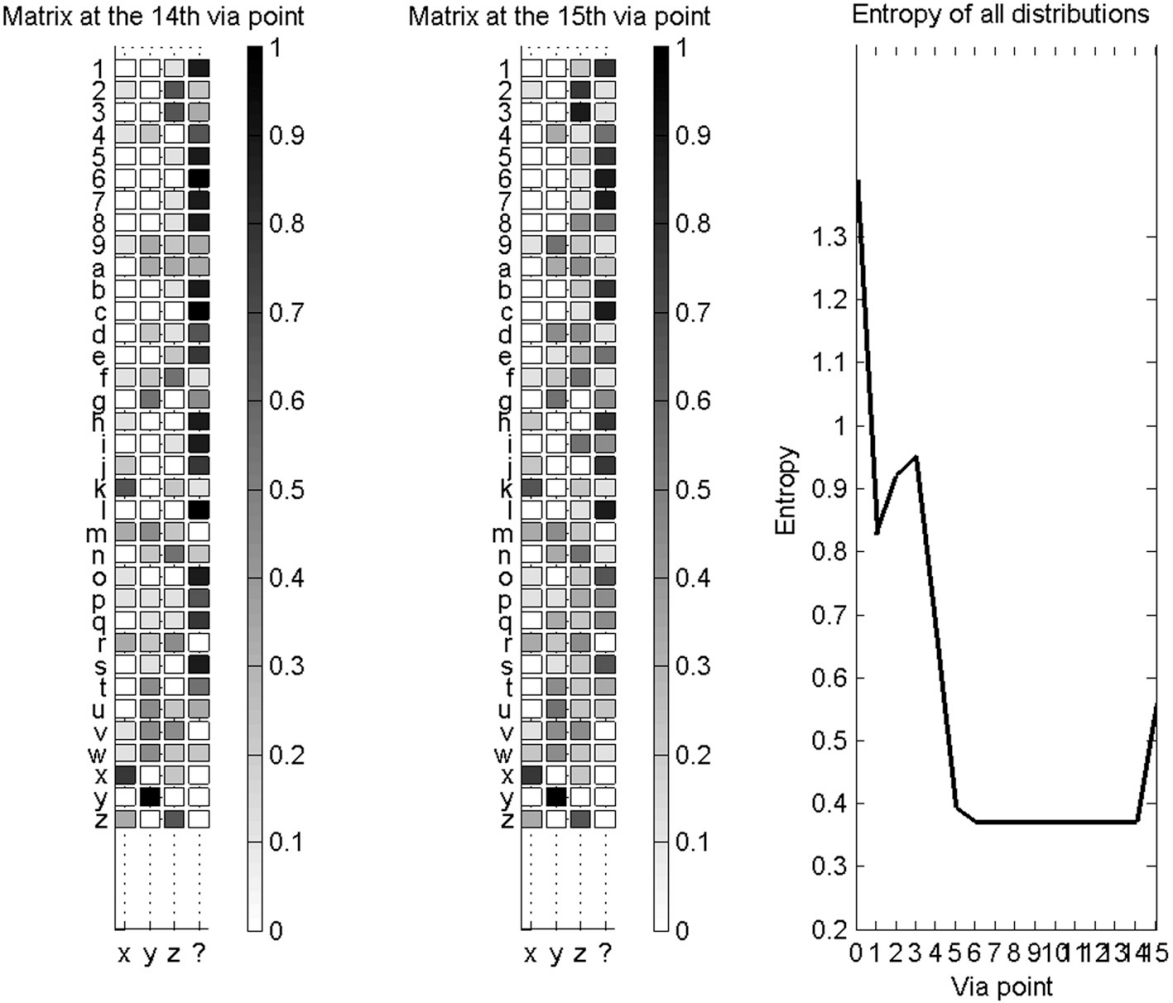
**Novelty detection for a database of known characters restricted to “x,” “y,” “z,” and the unknown character “$”. Left**: confusion matrix for the recognition of all available characters, after the collection of 14 via-points. **Middle**: confusion matrix for the recognition of all available characters, after the collection of 15 via-points and auxiliary variables. In both cases (left and middle), most characters are correctly recognized as new characters. **Right**: entropies of the distributions over letters, as via-points are accumulated. The addition of auxiliary variables makes the probability distributions more uncertain, in this case.

We also compute, as before, confusion matrices in this experimental context. They are not square matrices, because only 4 symbols can possibly be recognized, while we present 35 different test characters. Figure 13 shows confusion matrices, after the fourteenth via-point, and after the fifteenth via-point (which is accompanied by auxiliary variables), and the evolution of entropy of the probability distributions over letters as via-points are accumulated.

We observe that auxiliary variables reduce the capacity of the system to correctly detect novelty; for instance, the width and height of characters do not vary much over the whole alphabet, so that detecting a likely width and height increases the probability that the character is a known character. Therefore, we only let the system conclude that the presented trajectory is a new character if the probability of character “$” is high before auxiliary variables are measured.

We also observe that, although most characters are correctly recognized as unknown characters, some are incorrectly indentified as “x,” “y,” or “z.” This is due to our filtering process, where probability distributions are widely smeared around learned exemplars. This helps largely alleviate the scarcity of our learning database, but, on the other hand, this makes the recognition process so forgiving that novelty detection is impaired.

The accuracy and robustness of our letter recognition and novelty detection processes are clearly at odds, and the trade-off between these warrants further study. Unfortunately, it is likely that an optimal compromise would be elusive, and this trade-off would be application dependent. However, varying this compromise during adaptation to a new user is a promising direction. When a new user populates the learning database, the system would be sensitive to novelty, so that the database of learned letters grows. However, later on, when most symbols already are learned, the system would automatically become more robust, as the likelihood that the user would use a yet unseen character decreases.

### Disability assessment: experimental results

The final experiments we present concern the evaluation of motor characteristics of produced trajectories, independently of the represented letter, for disability assessment.

To do so, we simulate data from motor impairment loosely inspired by amyotrophic lateral sclerosis (ALS)^1^. So far indeed, all trajectories in our database have been provided by a healthy person, author Jean Lorenceau. Therefore, in the probabilistic model, these trajectories are associated with value [*H* = 1]: the *H* variable, representing disability levels, takes value 1 for healthy users, 2 for mildly impaired users, and 3 for heavily impaired users. We use the available trajectories and deteriorate them, with two different levels of manipulation, to generate two virtual databases, each of 35 * 8 characters, to associate to [*H* = 2] and [*H* = 3]. In order to simulate these disabled motor control levels, we imagine the following impairments.

Firstly, we assume that disability affects the size of produced trajectories, with reduced movement amplitude for increased disability level. Technically, from initial trajectories, we scale them down spatially by a factor 4/5 for [*H* = 2], and 1/2 for [*H* = 3]. Secondly, we add tremor movement to the initial trajectories, algorithmically simulated by adding a sinusoidal component of small amplitude for [*H* = 2] and of medium amplitude for [*H* = 3]. Technically, while this certainly displaces marginally some control points, this mostly affects the probability distribution about the high frequency components of the signal, *P*(*A*| *L, H*). Thirdly and finally, we assume that motor deterioration affects pupil control, with a slight increasing mean pupil diameter with increased disability level, and reduced pupil diameter variability.

**Figure 14:**
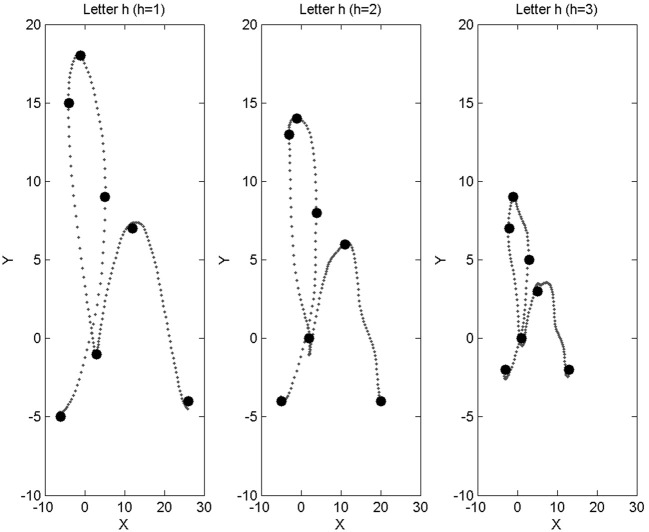
**Left:** example trajectory for the letter “h,” taken from our database, and associated with base disability level [*H* = 1] (healthy subject). **Middle, right**: trajectories obtained from the “h” on the left, after application of our algorithmic simulation of motor deterioration (**middle**: medium impairment, [*H* = 2]; **right**: large impairment, [*H* = 3]).

Figure 14 shows a letter “h” of our original database, along with trajectories obtained by our algorithmic simulation of increased disability: reduced size and added tremor are easily observed.

With our original and simulated trajectory databases, we learn parameters for the full BAP-EOL model, and use it to perform disability assessment. We consider all learned trajectories, and, for each, we feed them to the system and use Bayesian inference to compute:

$$P\text{​}\left(H\text{​}|C_{\Delta x}^{1:15},C_{\Delta y}^{1:15},C_{\dot{x}}^{1:15},C_{\dot{y}}^{1:15},S_{x},S_{y},A,\mu_{p},\sigma_{p}\right).$$

We thus build a 3 * 3 confusion matrix, the average of these probability distributions over variable *H*, for our complete database. It is shown Table 1.

**Table 1:**
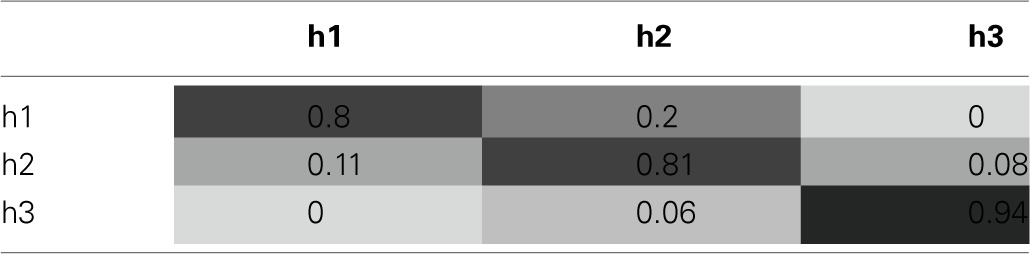
**Confusion matrix in the disability assessment experiment**.

*The table is read in rows. For instance, the top row is the average probability distribution over recognized disability level, for all trajectories of healthy users in the learning database. Diagonal values indicate correct recognition*.

In this experiment, we obtain a mean correct recognition score of 81%. Of course, our disability simulation uniquely determines this score. In other words, this experimental measure can be arbitrarily increased by simulating more separated levels of impairments, and, conversely, it can be arbitrarily decreased by simulating less distinct levels of impairment.

However, this still validates, as a proof of concept, the manner in which the flexibility of probabilistic modeling can be leveraged, in order to perform (general) disability assessment. Of course, a precise model of motor impairment would have to be developed for each disability and each patient population. Provided such a model can be acquired and included in the BAP-EOL model in lieu of our algorithmically simulated disability, computed probability distributions over variable *H* can be recorded as the user performs trajectories. These distributions can then be presented to the accompanying medical staff as a measure, to be interpreted and, if required, to be complemented by adequate diagnostic tests.

## Discussion

In this paper, we have presented the BAP-EOL model. It is a variant of a previous probabilistic model of isolated letter reading and writing, adapted to the original case of eye writing. In the model, letters are internally represented as sequences of via-points, along with auxiliary information about global size of letters, high-frequency components amplitude during movement production, and pupil diameter during trajectory tracing.

In this context, we have shown how the knowledge stored in the BAP-EOL probabilistic model could be manipulated, thanks to Bayesian inference, in order to solve three main tasks. Online letter recognition for instance, is based on computing probability distributions over letters, given an input trajectory. Experimental results show promising correct recognition score, even from a very scarce learning database.

The second task we solved was novelty detection: thanks to a probabilistic model of the unknown letter, character processing can recognize when the presented trajectory does not conform to learned letter shapes. Preliminary results, here, allowed exploring experimentally the tradeoff, in this process, between recognition robustness and novelty detection sensitivity.

The third and final task was disability assessment, in the context of eye writing by motor impaired patients. Indeed, trajectory recording is of course necessary for letter recognition, but it is also a rich opportunity, an open window into the user's motor control. We illustrated the way in which the BAP-EOL model could be expanded to include models of different levels of motor impairment, and Bayesian inference used to recognize the user's disability level. Preliminary results, based on an imagined, simulated database of motor impairments, illustrated the feasibility of the task. Application to ALS patients, and assessment of the benefit, if any, brought by this measurement tool (Gordon et al., 2012), are part of future work.

Of course, however promising these experimental results may be, they do not conceal the many open challenges that remain on the path to automatic processing of eye writing. For instance, the BAP-EOL model does not include an explicit method for dealing with via-point insertion and deletion. Via-points are a powerful summary of whole trajectories, but this representation is sensitive to the pairing of memorized via-points to the ones extracted from the input trajectory to be recognized. In particular, errors in this pairing at the start of trajectories may not be recovered from, and impact negatively character recognition.

Whereas beginnings of trajectories in handwriting are rather stereotyped, which alleviates somewhat this issue, it is not the case for eye writing. Indeed, using our apparatus, eye writing is performed thanks to an original sensory-motor loop: some smooth pursuit elicits perception of an illusory moving target which itself makes smooth pursuit easier, and reinforces the illusion, etc. We have therefore observed that the beginnings of trajectories often include intrusive saccades, or wandering, undirected smooth pursuit, before fluid letter tracing. This “burn-in” period, of movement initiation, often inserts superfluous via-points, which negatively interferes with character recognition. A specific probabilistic model of movement initiation, or a more robust system of via-point pairing, would help toward solving this issue.

Using the eye writing system to write words instead of isolated letters might also be another opportunity to reduce the impact of intrusive saccades related to movement initiation. In that case, which ultimately is closer to the general use case of the system, letter segmentation would be necessary. Our probabilistic model offers the opportunity, thanks to online letter recognition, to include prediction of the end of letter trajectories, to help letter segmentation. Preliminary work in this direction is promising, but warrants further developments.

Finally, it is also possible that the type of salient features we used, inherited from previous work concerning handwriting, is maladapted to the context of eye writing, and negatively impacts recognition scores. Zeroes in *x* and *y* velocity profiles have the advantage of being both geometrically salient and meaningful with respect to motor control, and seemed appropriate for the trajectories produced by author Jean Lorenceau. Alternate choices of features abound in the literature of handwritten character recognition, like detection of segmentation points between strokes, detection of velocity extrema, etc.

However, recent observations of additional subjects and the trajectories they produce during training indicate that alternate strategies of eye writing exist, with a larger departure from handwriting. Some subjects seem to produce mostly straight segments and sharp angles; others often include “burn-in” periods inside of letters, as they temporarily lose control of smooth pursuit, etc. Defining optimal features for eye written character recognition will have to follow a proper taxonomy of eye writing styles and their geometric properties. Such a taxonomy is yet to be obtained.

In this paper, we have highlighted a number of aspects by which handwriting and eye writing differ. The BAP-EOL model allows studying these differences; to the best of our knowledge, this is an original object of study. Additionally to this fundamental exploration of eye writing, our preliminary results hint at the feasibility of automatic trajectory processing for letter recognition and disability assessment.

Even in the case that letter eye writing would turn out to be inferior to existing methods for communication with motor impaired patients (e.g., if throughput is lesser with eye writing than with virtual keyboards), the eye writing system still would allow artistic free-flow production and drawing; we hope this might still be an invaluable tool to motor impaired patients, allowing them to continue expressing themselves in an emotionally rich manner, despite their motor disability.

### Conflict of interest statement

The authors declare that the research was conducted in the absence of any commercial or financial relationships that could be construed as a potential conflict of interest.

## Footnotes

^1^ALS patients will be the first test population that will have access to the eye writing system, later on in this research project.

## References

[B1] Anstis S. M. (1970). Phi movement as a subtraction process. Vis. Res. 10, 1411–1430 10.1016/0042-6989(70)90092-1 5516541

[B2] Artières T. Marukatat S. Gallinari P. (2007). Online handwritten shape recognition using segmental hidden markov models. IEEE Trans. Pattern Anal. Mach. Intell. 29, 205–217 10.1109/TPAMI.2007.38 17170475

[B3] Bolger C. Bojanic S. Sheahan N. F. Coakley D. Malone J. F. (1999). Dominant frequency content of ocular microtremor from normal subjects. Vis. Res. 39, 1911–1915 10.1016/S0042-6989(98)00322-8 10343779

[B4] Colas F. Diard J. Bessière P. (2010). Common Bayesian models for common cognitive issues. Acta Biotheor. 58, 191–216 10.1007/s10441-010-9101-1 20658175

[B5] Diard J. Gilet E. Simonin E. Bessière P. (2010). Incremental learning of Bayesian sensorimotor models: from low-level behaviours to large-scale structure of the environment. Connect. Sci. 22, 291–312 10.1080/09540091003682561

[B6] Gilet E. Diard J. Bessière P. (2011). Bayesian action–perception computational model: interaction of production and recognition of cursive letters. PLoS ONE 6:e20387 10.1371/journal.pone.0020387 21674043 PMC3106017

[B7] Gilet E. Diard J. Palluel-Germain R. Bessière P. (2010). Bayesian action-perception loop modeling: Application to trajectory generation and recognition using internal motor simulation, in Proceedings of the Thirtieth International Workshop on Bayesian Inference and Maximum Entropy Methods in Science and Engineering (Maxent 2010), eds Mohammad-Djafari A. Bercher J.-F. Bessière P. (Melville, NY: American Institute of Physics Conference Proceedings), 59–66

[B8] Gordon P. H. Salachas F. Bruneteau G. Pradat P.-F. Lacomblez L. Gonzalez-Bermejo J. (2012). Improving survival in a large french als center cohort. J. Neurol. 259, 1788–1792 10.1007/s00415-011-6403-4 22258480

[B9] Hu J. Brown M. K. Turin W. (1996). HMM based online handwriting recognition. IEEE Trans. Pattern Anal. Mach. Intell. 18, 1039–1045 10.1109/34.541414

[B10] Lebeltel O. Bessière P. Diard J. Mazer E. (2004). Bayesian robot programming. Auton. Robots 16, 49–79 10.1023/B:AURO.0000008671.38949.43 23012519

[B11] Lisberger S. G. Morris E. J. Tychsen L. (1987). Visual motion processing and sensory-motor integration for smooth pursuit eye movements. Annu. Rev. Neurosci. 10, 97–129 10.1146/annurev.ne.10.030187.000525 3551767

[B12] Lorenceau J. (2012). Cursive writing with smooth pursuit eye movements. Curr. Biol. 22, 1506–1509 10.1016/j.cub.2012.06.026 22840521

[B13] Martinez-Conde S. Macknik S. L. Hubel D. H. (2004). The role of fixational eye movements in visual perception. Nat. Rev. Neurosci. 5, 229–240 10.1038/nrn1348 14976522

[B14] Murphy K. (2006). Markov decision processes, learning of, in Encyclopedia of Cognitive Science (Hoboken, NJ: John Wiley and Sons, Ltd).

[B15] Plamondon R. Srihari S. N. (2000). On-line and off-line handwriting recognition: a comprehensive survey. IEEE Trans. Pattern Anal. Mach. Intell. 22, 63–84 10.1109/34.824821

[B16] Rehman A. Saba T. (2012). Off-line cursive script recognition: current advances, comparisons and remaining problems. Artif. Intell. Rev. 37, 261–288 10.1007/s10462-011-9229-7

[B17] Russell S. Norvig P. (1995). Artificial Intelligence: A Modern Approach. Englewood Cliffs, NJ: Prentice Hall Series in Artificial Intelligence

[B18] Sheahan N. F. Coakley D. Hegarty F. Bolger C. Malone J. F. (1993). Ocular microtremor measurement system: design and performance. Med. Biol. Eng. Comput. 31, 205–212 10.1007/BF02458038 8412372

[B19] Shiffrin R. M. Lee M. D. Kim W. Wagenmakers E.-J. (2008). A survey of model evaluation approaches with a tutorial on hierarchical bayesian methods. Cogn. Sci. 32, 1248–1284 10.1080/03640210802414826 21585453

[B20] Tappert C. C. Suen C. Y. Wakahara T. (1990). The state of the art in online handwriting recognition. IEEE Trans. Pattern Anal. Mach. Intell. 12, 787–808 10.1109/34.57669

[B21] Wakahara T. Murase H. Odaka K. (1992). On-line handwriting recognition. Proc. IEEE 80, 1181–1194 10.1109/5.156478

